# Malicious anchor node extraction using geodesic search for survivable underwater wireless sensor network

**DOI:** 10.1038/s41598-022-17956-9

**Published:** 2022-08-11

**Authors:** Prateek, T. Srinivasa Reddy, Saurabh Chandra, Rajeev Arya, Ajit Kumar Verma

**Affiliations:** 1 Wireless Sensor Networks Laboratory, Department of Electronics and Communication Engineering, National Institute of Technology Patna, Patna, Bihar 800005 India; 2 Faculty of Engineering and Natural Sciences, Western Norway University of Applied Sciences, Haugesund, Norway

**Keywords:** Electrical and electronic engineering, Computer science

## Abstract

Localization in underwater wireless sensor network (UWSN) faces an imminent threat when the triangulating anchor node starts to malfunction. Traditional geometric approaches are insufficient to cope with the survivability of UWSN topology. To address these issues, this paper presents a symplectic geometry for identification of the malicious anchor node. Consequently, a geodesic search algorithm (GSA) based Target localization is proposed which reduces the positioning error by exploiting the phase-space constancy of the underwater acoustic sensor network topology to effectively triangulate the target node despite its mobility. First, a malicious anchor node model is presented. The node movement is expressed in the form of “ripple region”. GSA is then proposed which effectively frees the node metastasis from anchor node geometry, thereby making the underwater system more survivable and resilient. Simulation results evaluate the survivability of the geodesic formalism in terms of the reduced penalty incurred by node movement, as well as the reduced impact of anchor node malfunction. An improvement of 13.46% and 9.26% reveals the utility of the geodesic technique in aquamarine sensor deployments, which would be beneficial in underwater resource exploration and defense planning.

## Introduction

Target localization is the science of determining the spatiotemporal placement of a target object for the purpose of surveillance, search and rescue operations, environmental monitoring and neighborhood sensing, etc. Because the position of target is initially unknown, the process of localization usually involves some kind of geometric reference. There are various geometric frameworks available in literature such as Euclidean geometry, trigonometric fundamentals, differential geometry, algebraic approach, computational geometry, etc. Each method has its own merits and field of application to real scientific scenarios. Some methods are suitable in terrestrial environments while others are applicable to aerial setups. An interesting application is the underwater domain, since 70% of the earth’s surface is covered with water. Any technique which could be suitably applied to underwater domain shall have far-reaching consequences. It shall prove to be of immense contribution to the scientific community, especially to underwater wireless sensor networks (UWSNs), which is the branch of wireless communication dealing with sensor nodes deployed in an underwater scenario. Symplectic geometry is a branch of differential geometry which deals with the curved mechanics of distance-traversing in a complicated topological space. Use of a symplectic geometry in a UWSN based target localization scenario is relatively scarcely explored. It shows the promise of approximating complicated multipaths such as Geodesic curvatures, which would otherwise be difficult to compute. Under adverse conditions, the survivability of any wireless communication system is of utmost importance. Some of the recent works in the literature pertaining to survivability of a UWSN are discussed in the related works section below.

## Related works

Survivability of a UWSN depends on the ability of underwater sensor nodes to operative despite external disruptions. External disruptions can be of various types, such as a torpedo attack in case of engagement of submarine networks, or a malicious anchor node affecting localization capability of UWSN, or autonomous underwater vehicle (AUV) getting displaced from its actual trajectory, etc. Accordingly, defense lines in networks are categorized into preventive, reactive and tolerance defense^1^. Survivability may be achieved through routing discovery, data transmission and key management. A planar engagement scenario is considered in^2^. A decoy launch management system is developed by taking the help of analytical justification as well as with simulations. Engagement planning is usually done to avoid getting hit by a torpedo^3^ in underwater warfare. An acoustic countermeasure system is crucial for multiple ships during torpedo evasion. To simulate such a scenario, requires an accurate setup of a combat environment as well as combat process. The movement model of torpedo, submarine and an acoustic decoy is presented in^4^. To deal with external disruptions of AUVs, the water flow around the AUV shape is profiled. The transient force and torque on AUV are analyzed to control the AUV hydrodynamics^5^. UWSN disruption may occur due to failure of cluster heads. By providing backup cluster heads^6^, a dependable clustering protocol is proposed which addresses the survivability issue of UWSN. Submarine Sensor Networks can be made more robust by testing different node geometries^7^ such as rectangular node geometry with two survivable nodes, or a rectangular geometry with round edges using multiple cables, or a rhombus shaped network topology. Multiple geometry has also been attempted to enhance survivability through the clever use of multiple decoy deployment in^8^. The parameters identified are the rate of turn, the field of view, the intercept time, etc.

Halt-scheduling is attempted with a load equilibrium mechanism to enhance lifespan of the UWSN in^9^. Stochastic modelling^10–15^ is an effective technique to ensure survivability of UWSN under varying conditions. Markov chain modelling^16,17^ is yet another established technique which has gained momentum in process calculus for underwater survivable sensor networks. In^10^, the relationship between group trust parameter and system lifetime is an indicator of survivability. The formulation of stochastic petri net (SPN) mathematical model evaluates this relationship. Authors have developed a mission effectiveness (ME) metric and a group communication system based on social networks. For mission success, a multi-dimensional trust system is considered. A Performance Evaluation Process Algebra (PEPA) workbench tool is used to verify the theoretical claims of stochastic process algebra in^11^. An overlay network is put up as a backup for survivability under network failure in^12^. A low power overlay network has also been proposed to overlap and to ensure survivability of a high resource sensor network^18^. Some parameters identified in^13^ related to Wireless Sensor Network (WSN) survivability are: frequency of failures, the data loss, the delay and the compromised data due to failures. Three types of failures identified are node failure due to power faults, link failure due to communication faults, and, attack failure due to black hole attacks. The authors have applied probabilistic model checking using tools such as Probabilistic Model Checker (PRISM) and continuous time Markov Chain (CTMC) model. Authors in^14^ have studied data integrity, which is directly proportional to data survival rate and inversely proportional to the location privacy. To estimate the location privacy, they propose three location estimation algorithms, namely the coordinate median, the average of overlapping area and the expectation maximization (EM). They show that the number of data replicas is inversely proportional to location privacy by using stochastic modelling. Stochastic model has also been proposed in^15^ to address the node isolation problem. Specifically, the authors have observed the topological survivability of k-connected networks. Similar to^14^, authors in^15^ have observed that the network survivability is inversely proportional to likelihood of node misbehaviors. A complex recovery process has been attempted using system repair model which consists of Markov Chain Matrix Exponential (ME) model, as presented in^16^. Similarly, stochastic models such as Markov Chain, semi-Markov process, reliability block diagrams and Markov reward models are analyzed for survivability and reliability in^17^.

Survivability in^19^ has been designed in terms of the number of sets (*k*) of WSNs which can cover a given area. It is shown that $k = 1$ means 1 set of WSN covers the region, whereas $k > 1$ indicates more than 1 set of WSN covering the region. A defensive resource allocation model^20^ using modified genetic algorithm is used to evaluate survivability index in a cyber physical power system (CPPS). The functional units of CPPS are proposed as atomic services. Graph theory to restructure dependencies in interdependent networks is applicable to HetNets as presented in^21^. A detailed algorithmic approach to combine network capacity with survivability is in^22^. Capacitated resilience parameter is compared with other reliability/survivability parameters such as k-terminal reliability, all-terminal reliability, traffic efficiency, and k-connectivity. Table 1 compares some of the latest localization works in the UWSN:

**Table 1:** Latest UWSN localization and geodesic works in literature.

Localization algorithm	Type of issue addressed	Method used
PDG-MMS^23^	Data gathering reliability	Priority clustering of mobile Sinks
STRING^24^	Low accuracy in DOA estimation	Information Geometry based scaling transform
Ref.^25^	UWSN architecture	Survey of UWSN localization
Face-based Gradient Optimization^26^	Slow convergence and requirement of linear system conventionally	Parallel gauss Seidel Solver
EEL-MDP^27^	Propagation delay in UWSN	Mobility pattern prediction, precise time synchronization, energy efficient localization
Learning by Optimization of a MIMO-Broadcast-Related Criterion over Space^28^	Issue of learning over complex -valued matrix hypersphere	Geodesic search sub-algorithm
Hybrid optimized localization technique^29^	High energy consumption, latency and error in traditional ranged localization	Hop-count based Time of Arrival (ToA) estimation method

The major problems identified in a UWSN localization are mentioned as follows:

- *Lack of literature on underwater geodesic framework*: The issue of multipath propagation in underwater acoustic communication has severe implications. Traditional propagation models fail to accurately represent the acoustic propagation in marine and stratified waters. Since Euclidean geometry has a limited scope in such a scenario, hence there is a strong need for evaluation of geodesic framework which would enable measurements across manifolds to accurately pinpoint the target.
- *Survivability of UWSN under node metastasis*: Underwater sensor nodes which are constantly drifting are a source of error during target localization. Survivability of a UWSN is jeopardized if it cannot make up for the sensor node metastasis. It is crucial to determine the points of failure to ensure that the UWSN survives the adversities.
- *Lack of malicious anchor node models for UWSN*: Malicious anchor nodes could be a threat to the underwater target localization. To the best of authors’ knowledge, the combined effect of malicious anchor node with sensor node metastasis has not been analyzed in the underwater setting. There is an urgent need to formulate and comment upon the malicious behavior using a geodesic frame of reference.

The present work serves as a starting point to address these vital issues at hand. The major contributions of this paper are as follows:

- The concept of malicious anchor node in underwater acoustic sensor network is fused with the node metastasis through the phase-space representation of ripple region.
- A geodesic formalism is proposed under the umbrella of geodesic search algorithm to separate malicious node effects from the anchor node topology.
- For stretch-ripple region condition, the key parameters such as ripple region penalty, percentage node metastasis and normalized malicious node content are evaluated to gauge the effectiveness of the proposed geodesic technique.

The rest of the paper is organized as follows: “Section Malicious anchor node model” presents a malicious anchor node model and discusses the significance of ripple region in underwater scenario. “Section Geodesic search algorithm” frames a geodesic search algorithm (GSA) to incorporate symplectic geometry into the UWSN topology. “Section Simulation results” evaluates the performance of the proposed technique with respect to different parameters and compares it to the standard results for verification of the proposed technique. Final words are summarized in “Section Conclusion”.

## Malicious anchor node model

In this section, a scenario is defined in which a discrete grid of underwater acoustic nodes is spatially and temporally distributed. One anchor node is not working the way it is supposed to. Since the target is to be localized with the reference of the known coordinates of the anchor nodes, therefore, a malicious anchor node Model is defined so that the loss of accuracy due to malicious anchor node may be computed and compensated for.

**Figure 1:**
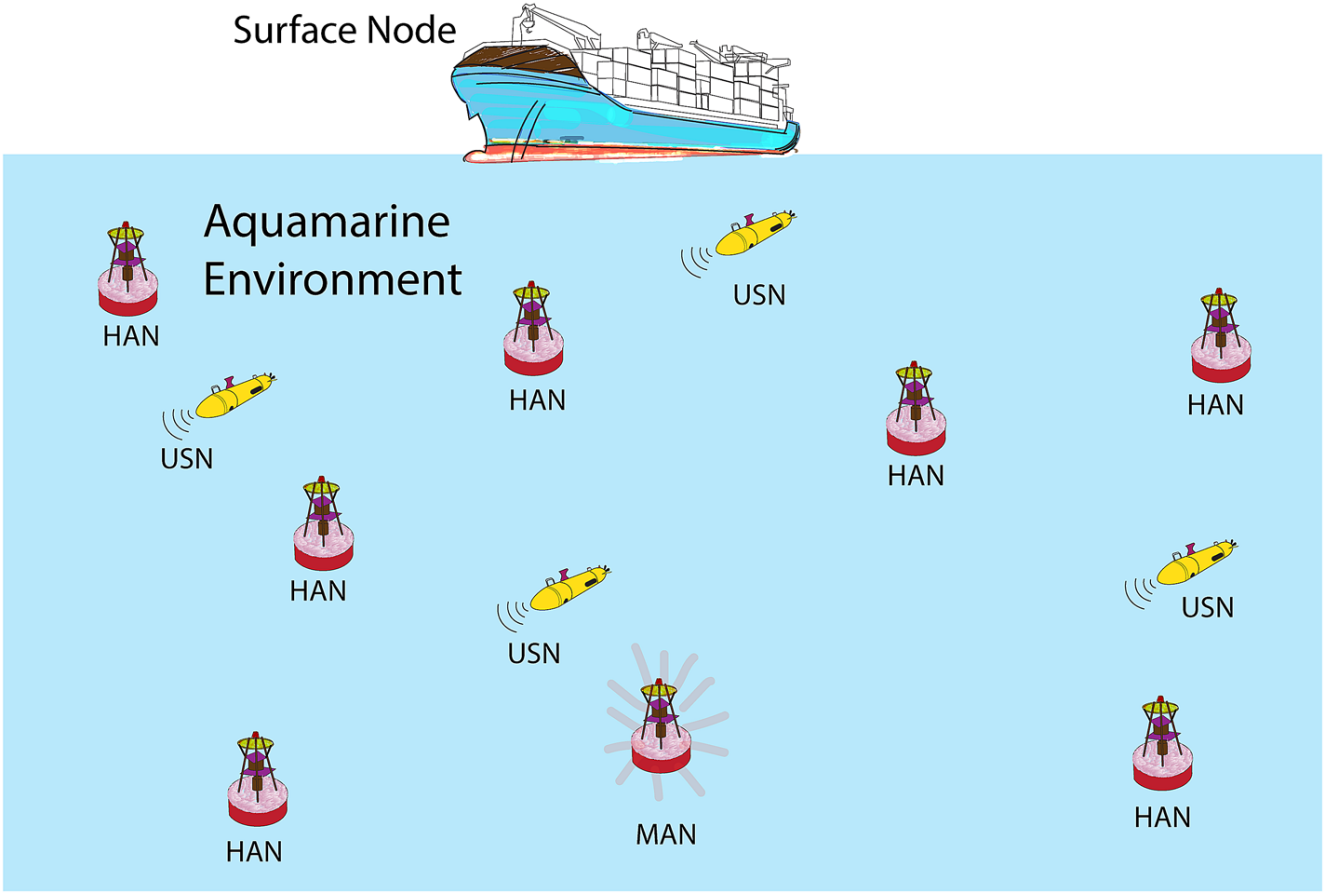
An underwater wireless sensor network scenario. HAN, Healthy Anchor Nodes; MAN, Malicious Anchor Node; USN, Underwater Sensor Node.

Let the underwater anchor and sensor node network be represented by a symplectic form in a non-turbulent underwater scenario, whose manifold $M$ be differentiable. Let $C$ be a non-degenerate, lower-dimensional manifold. In a family of acoustic vector nodes $\mu$, a malicious anchor node is defined as the one which either stores the wrong coordinate information about itself, or it claims to be in the vicinity of the target even though the target is beyond the connectivity range of the anchor node. The UWSN scenario is presented in Fig. 1. The ship represents a surface node. Healthy Anchor Nodes (HAN) are scattered and guide the underwater sensor nodes (USNs). The USNs are subject to metastasis constantly. The Malicious Anchor Node (MAN) exhibits malicious behavior, disrupting the smooth operation of the UWSN topology.

### Definition 3.1

(Malicious anchor node) An anchor node $c \in C$ is a non-malicious anchor node of a sensor network $\mu$ if there is a connectivity region $\cup$ of $c \in C$ such that, for all subsets $u \in \cup$ of the intended connectivity region, $\mu \left( u \right)$ and $\mu \left( c \right)$ convey the same topological information as the other anchor nodes. An anchor node $c \in C_{B}$ is a malicious anchor node of $\mu$ if it conveys different topological information. Let $\mu \left( {C_{B} } \right)$ denote the set of malicious anchor nodes of $\mu$. The percentage of malicious anchor nodes is given by the expression

1 $$\frac{{\mu \left( {C_{B} } \right)}}{{\mu \left( C \right) + \mu \left( {C_{B} } \right)}} \times 100,$$

where $\mu \left( C \right)$ represents the set of non-malicious anchor nodes, and $\mu \left( {C_{B} } \right)$ is the set of malicious anchor nodes.

In any underwater acoustic node localization, the drifting behavior may either bring the node topology closer together, or it may disperse them farther away. In either case, dislocation of the sensor network from its intended position shall introduce localization errors. The usual plan of action remains to model the sensor network behavior, then to minimize these errors to below the tolerable limits. The drifting behavior is defined in terms of a Ripple Region, as follows:

### Definition 3.2

(Ripple Region) A ripple region is characterized by spatial region where the initial phase-space is different from final phase space.

### Significance of ripple region

**Figure 2:**
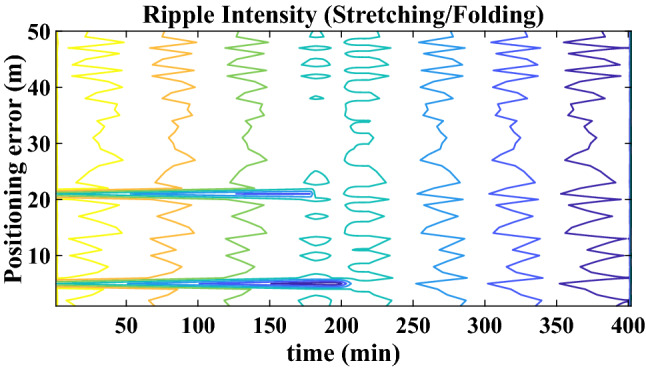
Phase space in terms of Ripple Region Intensity.

Let the initial phase-space be denoted by $g_{u.p} \in {\mathbb{R}}^{2}$. For simplicity, a two-dimensional spatial region is considered, though, the concept may be extended suitably to higher dimensions too. The final phase-space is then given as $g_{p} \in {\mathbb{R}}^{2}$, as shown in Fig. 2. The anchor nodes denoted by $\overline{a}_{i}$ and the sensor nodes denoted by $\overline{s}_{i}$ float in water and are subjected to ravages of underwater nature. Due to the fluid nature of water, sensor nodes which are free-floating (that is, untethered to seabed or surface vessel) are influenced by water current. They could either float away or float inwards. In the first case, the sensor network diverges from its initial topology, whereas in the second case, the sensor network bunches up together.

Summarizing “Section Malicious anchor node model”, a Malicious anchor node model consists of a system of acoustic anchor nodes surrounding the unknown target node, of which one or more anchor nodes are behaving maliciously. Being deployed underwater, the nodes are not stationary, therefore, the node current has been expressed into the anchor node model by a pattern which represents the effect where the anchor nodes diverge from the target resulting in a distinct geometrical topology. In the coming sections, this topological pattern shall be exploited to search for the positioning information of the unknown target node.

## Geodesic search algorithm

The proposed technique named “geodesic search algorithm” is described in this section. It is a technique which separates ripple region from anchor node geometry, as per proposition 4.1 mentioned below.

### Proposition 4.1

Let $\overline{f}$ be the curve representing the scope of the ripple region in a uniform phase-space. Assume that $\overline{f}$ has an anchor node position $\overline{p}$ such that $\overline{f}^{\prime}\left( {\overline{p}} \right)$ represents the slope of the ripple region boundary and $\overline{f}^{\prime\prime}\left( {\overline{p}} \right)$ is the rate of change of ripple region boundary. If $\lambda_{1}$ and $\lambda_{2}$ are the eigenvectors corresponding to true anchor node and malicious anchor node, then the anchor node geometry becomes independent of the Ripple Region, provided that the Ripple Region contains $\overline{p}$.

Let us introduce the concept of “Geodesic norm” by equation (2)

2 $$\left\| {u,v} \right\|: = \sqrt {\left\langle {u,u} \right\rangle + \varepsilon \left\langle {v,v} \right\rangle }$$

where $u,v$ belong to manifolds $T_{p} ,M$ and some function $\varepsilon < \frac{1}{K}$, where $K$ is the connectivity of UWSNs.

$\frac{{\left\langle {Y,\dot{Y}} \right\rangle }}{{\left\| {Y,\dot{Y}} \right\|^{2} }} \ge \delta$ defines the propagation of acoustic signal traversing in underwater domain.

Let us define a lemma that restricts the extent of signal variation in an underwater scenario:

### Lemma 4.2

For Metastasis $$\delta$$ which is limited to $$\delta < \frac{K}{{1 + K^{{{\raise0.5ex\hbox{$\scriptstyle { - 3}$} \kern-0.1em/\kern-0.15em \lower0.25ex\hbox{$\scriptstyle 2$}}}} }}$$, the family of acoustic trajectory $$C_{\delta }$$ given by $$\frac{{\left\langle {Y,\dot{Y}} \right\rangle }}{{\left\| {Y,\dot{Y}} \right\|^{2} }} \ge \delta$$ is strictly immune to malicious effects.

It would be easier to prove this only for No Metastasis of anchor node, but we need some positive metastasis $\delta$ later to dwell upon the acoustic localization in presence of malicious anchor nodes.

### Proof

For some $\varepsilon$ and $k \in K$,

3 $$\frac{{\sqrt {\left\langle {Y,Y} \right\rangle \left\langle {\dot{Y},\dot{Y}} \right\rangle } }}{{\left\| {Y,\dot{Y}} \right\|^{2} }} \le \frac{1}{2\sqrt \varepsilon }$$

4 $${\text{and}}\quad \frac{{\left\langle {\dot{Y},\dot{Y}} \right\rangle + k\left\langle {Y,Y} \right\rangle }}{{\left\| {Y,\dot{Y}} \right\|^{2} }} \ge k$$

From Cauchy–Schwarz Inequality, and by limiting the metastasis $\delta$ to less than 0.05, we get

5 $$\frac{d}{dt}\left( {\frac{{\left\langle {Y,\dot{Y}} \right\rangle }}{{\left\| {Y,\dot{Y}} \right\|^{2} }}} \right) = \frac{{\left( {\left\langle {\dot{Y},\dot{Y}} \right\rangle + \left\langle {\ddot{Y},Y} \right\rangle } \right)\left\| {Y,\dot{Y}} \right\|^{2} - 2\left\langle {Y,\dot{Y}} \right\rangle \left( {\left\langle {Y,\ddot{Y}} \right\rangle + \varepsilon \left\langle {\ddot{Y},\dot{Y}} \right\rangle } \right)}}{{\left\| {Y,\dot{Y}} \right\|}}$$

Therefore, the first order derivative of the unit norm of $Y$ and $\dot{Y}$ is positive when Metastasis $\delta$ equals the unit norm of $Y$ and $\dot{Y}$. □

Lemma 4.2 shall be useful when proving that the proposed UWSN localization technique separates the dependence of ripple region on anchor node topology.

### Geodesic representation of the UWSN topology

To delve into this section using the symplectic geometry developed for underwater sensor networks, a function called “Geodesic formalism” representing the phase-space distribution is introduced here.

(A) Geodesic Formalism and stretch Ripple Region

The Geodesic concept is widely used in the domain of image processing to map a curved surface for specific display of perspective. Unlike L2 norm which maps the distance between two points as a straight line, the underwater positioning system requires a more sophisticated form of acoustic trajectory measurement, especially when dealing with stratified sound speed profile.

Let $A$ denote the set of anchor nodes amongst a subset of all the deployed sensor nodes ${\mathbb{R}}^{p}$. It is assumed throughout this work that for all anchor nodes $\overline{a} \in A$, the noise profile is independent of their physical location, that is, all anchor nodes face equal amount of noise irrespective of their actual position in the topology. Let $\left\| x \right\|_{G}^{{\overline{a}}}$ denote the geodesic norm of anchor node $\overline{a}$ from the target. The geodesic norm $\left\| x \right\|_{G}^{{\overline{a}}}$ is, thereby, given as

6 $$\left\| x \right\|_{G}^{{\overline{a}}} = \inf \left\{ {t > 0:x \in \sum\limits_{count\left( A \right)} {\left( {\frac{{\partial {\rm X}}}{\partial A}} \right)} } \right\}$$

where $t$ is the time instance of measurement, $count\left( A \right)$ is the number of anchor node hops taken by the acoustic signal to reach the target, and ${\rm X}$ is the total distance traversed by the acoustic signal when its path is considered as a continuous signal.

The issues of Malicious Node on anchor node information are a topic of analysis of their own. For now, let us restrict ourselves to the penalty incurred due to the stretch Ripple Region under the framework of Geodesic Formalism. Let a linear measurement model of intensity of stretch $\Phi$ for a simple Ripple Region be represented by $x^{ * }$, which is the dual of geodesic norm for the set of anchor nodes $A$. If the signal model is

7 $$\overline{y} = \Phi \overline{x}^{ * }$$

Then, the estimated geodesic norm $\widehat{{\overline{x}}}$ is computed from $\overline{x}$ such that

8 $$\widehat{{\overline{x}}} = \arg \min \left\| x \right\|_{G}^{{}}$$

9 $${\text{Subject}}\,{\text{to}}\quad \overline{y} = \Phi \overline{x}$$

A convex formulation may be easily obtained by relaxing constraint in (9) to $\left| {\overline{y} - \Phi \overline{x}} \right| < \Delta_{G}$, where $\Delta_{G}$ is the tolerable Geodesic noise floor. Subsequently, we state and prove the condition under which the Geodesic Formalism provides a unique and optimal solution to the anchor node topology under stretch ripple region.

#### Proposition 4.3

The dual of the Geodesic norm $x^{ * }$ equals the estimated geodesic norm $\hat{\overline{x}}$ and this estimated geodesic norm $\hat{\overline{x}}$ is the matched acoustic trajectory from the source anchor node to the target if and only if

10 $$E\left( {Y\left| {T^{ * } = t} \right.} \right) = E\left( {h\left( {X_{1} \left( t \right), \ldots ,X_{n - 1} \left( t \right),\Phi \left( {t, \cup } \right)} \right)} \right)$$

where $X_{i} \left( t \right)$ are independent random variables uniformly distributed and independent of connectivity region $\cup$, and $\cup$ has density $g\left( {u\left| t \right.} \right)$.

#### Proof

$$\begin{aligned} & E\left( {Y\left| {T^{ * } = t} \right.} \right) \\ & \quad = E\left( {h\left( {X_{1} , \ldots ,X_{n} } \right)\left| {T^{ * } = t} \right.} \right) \\ & \quad = E\left( {h\left( {X_{1} , \ldots ,X_{{\left( {n - 1} \right)}} ,X_{n} } \right)\left| {T^{ * } = t} \right.} \right) \\ \end{aligned}$$

where $E\left( \cdot \right)$ is the expectation operator. By Crofton’s Theorem^30^, conditional on $T^{ * } = t$, the terms $X_{1} , \ldots ,X_{{\left( {n - 1} \right)}}$ have the same distribution as the generalized order statistics of $X_{1} \left( t \right), \ldots ,X_{{\left( {n - 1} \right)}} \left( t \right)$ as in the proposition 4.3 and $X_{\left( n \right)}$ is distributed as $\Phi \left( {t, \cup } \right)$. By symmetry of $h$, the result follows that the geodesic norm of the true measurement shall equal the geodesic norm of the estimated measurement. In other words, localization using geodesic formulation is feasible in case of UWSN as well.□

In order to model the behavior of Ripple Region stretching outwards, we first have to relate the support lines of the anchor nodes forming a convex space. Let $O_{1}$ denote the proximity of anchor node $a_{1}$ while $O_{2}$ be the proximity of $a_{2}$. Let the anchor nodes have support lines $A_{1} P$ and $A_{2} P$ such that $P$ denotes the point of intersection of the two supports, and $P$ be the location of the malicious node. Let $\tau_{1}$ and $\tau_{2}$ denote the vectors of support lines; change of angle $\omega$ indicates Ripple Region stretching ($\omega$ increasing) or folding ($\omega$ decreasing). In either case, the relationship between non-malicious and malicious anchor nodes would be critical to locating the target accurately. The Geodesic Formalism is explained below:

- Knowledge of $\left( {x,y} \right)$, $\sin \phi$,$\cos \phi$, distance $p$.
- To express $x$ and $y$ in terms of geometric coordinates.
- To express $dx$ and $dy$, and subsequently estimate the extent of perturbation caused by the malicious anchor node.
- To estimate the loss of information.
- To discretize (curved) acoustic measurement and determine the Geodesic norm to remove the Malicious effects.
- Optimal underwater localization.

According to Crofton’s Formula^31^, the position $\left( {x,y} \right)$ of the malicious node is given by

11 $$x\cos \phi_{1} + y\sin \phi_{1} - A_{1} P = 0$$

12 $${\text{and}}\quad x\cos \phi_{2} + y\sin \phi_{2} - A_{2} P = 0$$

Differentiating (11) with respect to $\Phi$, we get

13 $$\left( {A_{1} P} \right)^{\prime } = y\cos \phi - x\sin \phi$$

Then, (11) is rewritten as

14 $$\Rightarrow x\cos \phi_{1} = \left( {A_{1} P} \right) - y\sin \phi_{1}$$

$$\begin{aligned} & \Rightarrow x\cos^{2} \phi_{1} = \left( {A_{1} P} \right)\cos \phi_{1} - y\sin \phi_{1} \cos \phi_{1} \\ & \Rightarrow x\left( {1 - \sin^{2} \phi_{1} } \right) = \left( {A_{1} P} \right)\cos \phi_{1} - y\sin \phi_{1} \cos \phi_{1} \\ & \Rightarrow x = \left( {A_{1} P} \right)\cos \phi_{1} - y\sin \phi_{1} \cos \phi_{1} + x\sin^{2} \phi_{1} \\ & \Rightarrow x = \left( {A_{1} P} \right)\cos \phi_{1} - \left( {y\cos \phi_{1} - x\sin \phi_{1} } \right)\sin \phi_{1} \\ \end{aligned}$$

15 $$\Rightarrow x = (A_{1} P)\cos \phi_{1} - \left( {A_{1} P} \right)^{{\prime }} \sin \phi_{1}$$

Similarly, for (12)

16 $$\Rightarrow x = \left( {A_{1} P} \right)\cos \phi_{2} - \left( {A_{1} P} \right)^{{\prime }} \sin \phi_{2}$$

y coordinate is similarly computed as

17 $$y = \left( {A_{1} P} \right)\sin \phi_{1} + \left( {A_{1} P} \right)^{{\prime }} \cos \phi_{1} \quad {\text{for}}\,{\text{anchor}}\,{\text{node}}\,A_{1}$$

18 $$\& \quad y = \left( {A_{2} P} \right)\sin \phi_{2} + \left( {A_{2} P} \right)^{{\prime }} \cos \phi_{2} \quad {\text{for}}\,{\text{anchor}}\,{\text{node}}\,A_{2}$$

Next, the extent of perturbation of malicious node maybe parametrically expressed for x-coordinate as

19 $$x = A_{1} P\cos \phi_{1} - \left( {A_{1} P} \right)^{\prime } \sin \phi_{1}$$

$$\Rightarrow dx = - A_{1} P\sin \phi_{1} d\phi_{1} - \left( {A_{1} P} \right)^{{\prime }} \cos \phi_{1} d\phi_{1} + \left( {A_{1} P} \right)^{{\prime }} \cos \phi_{1} d\phi_{1} - \left( {A_{1} P} \right)^{{\prime \prime }} \sin \phi_{1} d\phi_{1}$$

$$\Rightarrow dx = - \left( {\left( {A_{1} P} \right) + \left( {A_{1} P} \right)^{\prime \prime } } \right)\sin \phi_{1} d\phi_{1} \quad {\text{according}}\,{\text{to}}\,{\text{anchor}}\,{\text{node}}\,A_{1}$$

$${\text{And}} \Rightarrow dx = - \left( {\left( {A_{2} P} \right) + \left( {A_{2} P} \right)^{{\prime \prime }} } \right)\sin \phi_{2} d\phi_{2} \quad {\text{according}}\,{\text{to}}\,{\text{anchor}}\,{\text{node}}\,A_{2}$$

Similarly, the extent for perturbation for y-coordinate is given from derivative of (17) as

20 $$\Rightarrow dy = A_{1} P\cos \phi_{1} d\phi_{1} - \left( {A_{1} P} \right)^{\prime } \sin \phi_{1} d\phi_{1} + \left( {A_{1} P} \right)^{\prime } \sin \phi_{1} d\phi_{1} + \left( {A_{1} P} \right)^{\prime \prime } \cos \phi_{1} d\phi_{1}$$

$$= \left( {A_{1} P} \right)\cos \phi_{1} d\phi_{1} + \left( {A_{1} P} \right)^{\prime \prime } \cos \phi_{1} d\phi_{1}$$

$$\Rightarrow dy = \left( {\left( {A_{1} P} \right) + \left( {A_{1} P} \right)^{\prime \prime } } \right)\cos \phi_{1} d\phi_{1} \, {\text{from anchor node}}\,A_{1} \,{\text{perspective}},$$

$${\text{and}},\quad \Rightarrow dy = \left( {\left( {A_{2} P} \right) + \left( {A_{2} P} \right)^{{\prime \prime }} } \right)\cos \phi_{2} d\phi_{2} \, {\text{from anchor node}}\,A_{2} \,{\text{perspective}}.$$

$dx$ and $dy$ respectively indicate the consensus in malicious anchor node positioning. Assuming that the movement of anchor node topology is smooth, the Phase-space of the geometry is approximated by the proposed Geodesic Formalism.

## Simulation results

For the purpose of computational evaluation of the proposed geodesic search algorithm (GSA) based on the Geodesic Formalism mentioned in “Section Geodesic search algorithm”, simulation is carried out for an underwater scenario using MATLAB. Popular inbuilt toolboxes such as signal processing toolbox, statistics & machine learning toolbox, global optimization toolbox, Communications System Toolbox etc. have been used. The key parameters evaluated are mentioned in brief as follows:

A. Ripple region penalty: Ripple region penalty can be defined as the amount of positioning error introduced into the system due to the lack of compensation of ripple intensity. The task of any localization algorithm becomes to compensate for the ripple intensity, consequently reducing the possibility of error in positioning. The expression for calculating ripple region penalty (R.R.P) is given by
  21 $$R.R.P = \frac{{E\left( {\left. Y \right|T^{*} = t} \right) - E\left( {h\left( {X_{1} \left( t \right), \ldots ,X_{n - 1} \left( t \right),\Phi \left( {t, \cup } \right)} \right)} \right)}}{{\left| {\overline{y} - \Phi \overline{x}} \right| + \Delta_{G} }} \times 100$$
  where the numerator is the penalty due to acoustic mismatch arising from the trajectory from the source node to the target node, as outlined in (10), and the denominator is the sum of the estimation error due to ripple region and the geodesic noise floor as explained in (9). A positive numerator denotes the stretched ripple region whereas a negative numerator means bunched-up ripple region. Ripple region may be calculated when there are stationary nodes, represented by the legend “proposed GSA”, or mobile nodes^32^ as shown by the legend “GSA+ Node Mobility”.
B. Percentage node metastasis: Another issue which plagues an underwater sensor network is the effect of separation distance between the stationary nodes from the non-malicious anchor nodes, on the extent of node metastasis. Therefore, the normalized node metastasis is computed using the proposed GSA for different levels of distances from stationary position of the node topology. The general expression for computation of percentage node metastasis is given by
  22 $$\delta \left( {\overline{f}} \right) = \frac{{f^{\prime\prime}_{1} \left( {\overline{p}_{1} } \right) - f^{\prime\prime}_{2} \left( {\overline{p}_{2} } \right)}}{{f^{\prime}_{1} \left( {\overline{p}_{1} } \right) - f^{\prime}_{2} \left( {\overline{p}_{2} } \right)}} \times 100$$
  where the numerator represents the difference between the rate of change of the slopes of ripple region boundary for one anchor node position and that of another anchor node position. The denominator represents difference of slopes of the ripple region boundaries corresponding to the two respective anchor nodes.
C. Normalized malicious node content: The number of malicious anchor nodes in the system heavily influences how the proposed technique counters the malicious node content with every successive round of measurement. Therefore, the rate of compensation of malicious node in UWSN holds significance of its own. The expression for the percentage node metastasis is given by
  23 $${\text{Node}}\,{\text{Metastasis}}\,(\% ) = \frac{{\mu \left( {C_{B} } \right)}}{{\mu \left( C \right) + \mu \left( {C_{B} } \right)}} \times 100$$
  where the symbols have meaning as mentioned in equation (1).

The numerical computations of the proposed technique are compared to some of the standard localization methods based upon survivability tactics. Decoy based localization is one such technique, where the target is localized after deployment of a decoy system. The decoy system increases the chances of survival of the underwater sensor network topology, which in turn enables higher probability of accurate positioning. Decoy based localization has been attempted in^9^ in the form of lifetime enhancement using halt-scheduling mechanism, link-failure analysis in^13^, graph theoretical approach in^19^ etc. In the following subsection, a detailed discussion regarding the above-mentioned key parameters shall be carried out. Table 2 outlines the broad values for the parameters used in the GSA Model.

**Table 2:** Parameters of GSA model.

Parameter	Value
Unstratified Sound Speed underwater	1500 m/s
Sound Speed Profile (stratified)	Ref.^33^
Maximum depth	11,000 m
Frequency of operation	2–4 kHz
Maximum acoustic node separation	500 m
Negative effects considered	Ripple Region, Node metastasis, malicious anchor node
Number of iterations	1000

### Discussion

**Figure 3:**
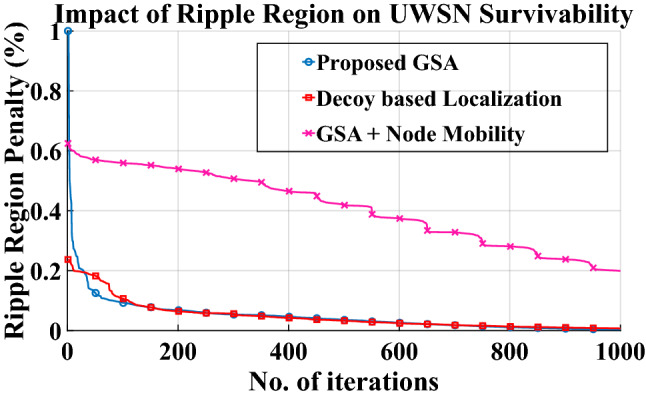
Impact of Ripple region on UWSN survivability.

**Figure 4:**
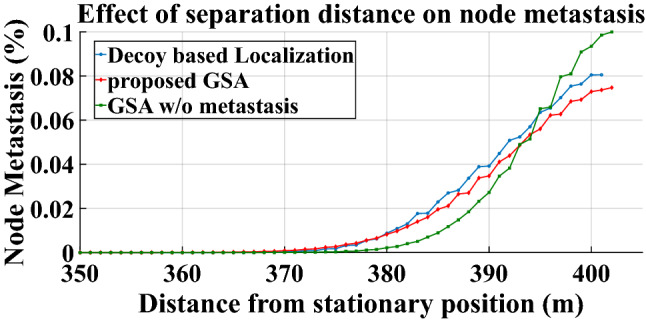
Effect of separation distance on node metastasis.

A. Ripple region penalty
  Ripple region penalty for the proposed scheme and the compared methods is shown in the Fig. 3. After 31 number of iterations, the proposed GSA based localization trumps the traditional Decoy-based localization. The proposed method exhibits 33% and 13.46% lower ripple penalty than the competing method at 50 iterations and 100 iterations, respectively. GSA achieves lower penalty because of its ability to measure acoustic trajectory along the geodesic lines instead of the straight Euclidean lines.
B. Percentage node metastasis
  Figure 4 depicts the effect of separation distance from stationary position on percentage node metastasis. Survivability of the UWSN topology depends on the minimization of anchor node metastasis. Since any algorithm that contains some form of metastasis-countering ability is better suited to an UWSN scenario, therefore the proposed GSA technique would perform poorly without any metastasis counter mechanism, as observed in the legend named “GSA w/o metastasis”, especially for distances larger than 395 m. Upon incorporating metastasis-countering steps into the GS algorithm, the extent of node metastasis is contained below the competing Decoy based localization for the entire range beyond 380 m. On an average, the GS algorithm (shown by the legend “proposed GSA”) is 9.26% better than the decoy-based method at a distance of 400 m. This performance may be attributed to the ability of the geodesic search to separate the effect of node metastasis from anchor node geometry.
C. Normalized malicious node content

**Figure 5:**
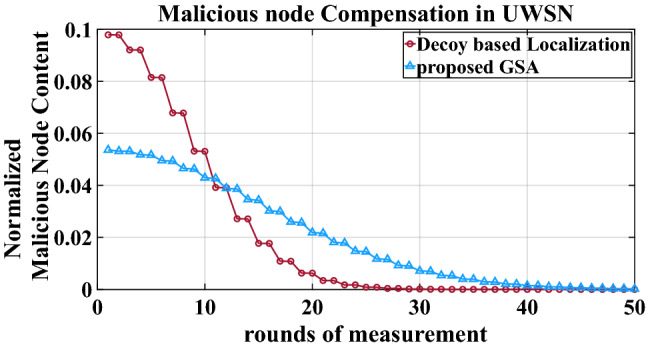
Malicious node compensation in UWSN.

The compensatory behaviour of the localization algorithms against the malicious nodes in UWSN can be seen through the lowering of normalized malicious node content with every successive round of measurement. Figure 5 demonstrates that initially, the decoy-based localization exhibits higher malicious node content. The point of inflexion between the proposed GSA and the decoy-based method is observed at the eleventh round of measurement. It indicates that under energy constrained scenario, the proposed method presents nearly 50% lower malicious node content that the competing method.

## Conclusion

The formalism presented here gave a fresh outlook towards visualizing the impact of geodesic concept on the survivability of the UWSN topology when inflicted with a malicious anchor node. The lack of literature on underwater geodesic framework was addressed with the help of the proposed GSA method. Survivability of the UWSN was explored under node metastasis condition. The lack of malicious anchor node model was supplemented with a Ripple-Region formulation. Preliminary results showed that the penalty due to ripple region was mitigated by at least 13.46%, whereas the metastasis due to node movement was suppressed by 9.26%. The proposed work further promised 50% lower malicious node content at moderate rounds of measurements. This established the feasibility of geodesic modelling to UWSN localization, and could be further explored in sparse sensor networks in acoustic aquamarine environments.

## Data Availability

All data generated or analysed during this study are included in this published article.
